# Spectroscopic insight into breast cancer: profiling small extracellular vesicles lipids via infrared spectroscopy for diagnostic precision

**DOI:** 10.1038/s41598-024-59863-1

**Published:** 2024-04-23

**Authors:** Abhay Mishra, Sadaqa Zehra, Prahalad Kumar Bharti, Sandeep R. Mathur, Piyush Ranjan, Atul Batra, Krishna K. Inampudi, Gyan Prakash Modi, Fredrik Nikolajeff, Saroj Kumar

**Affiliations:** 1 Department of Biophysics, All India Institute of Medical Sciences, New Delhi, 110029 India; 2 Department of Health, Education, and Technology, Lulea University of Technology, 97187 Luleå, Sweden; 3 Department of Pathology, All India Institute of Medical Sciences, New Delhi, 110029 India; 4 Department of Surgical Disciplines, All India Institute of Medical Sciences, New Delhi, 110029 India; 5 Department of Medical Oncology, All India Institute of Medical Sciences, New Delhi, 110029 India; 6 Department of Pharmaceutical Engineering & Technology, Indian Institute of Technology (BHU), Varanasi, 221005 Uttar Pradesh India

**Keywords:** sEVs, FTIR, Spectral marker, PCA, Lipid, Proteins, Carbohydrates, Nucleic acids, Breast cancer, Lipids, Infrared spectroscopy, Biomarkers

## Abstract

Breast cancer, a leading cause of female mortality due to delayed detection owing to asymptomatic nature and limited early diagnostic tools, was investigated using a multi-modal approach. Plasma-derived small EVs from breast cancer patients (BrCa, n = 74) and healthy controls (HC, n = 30) were analyzed. Small EVs (n = 104), isolated through chemical precipitation, underwent characterization via transmission electron microscopy (TEM) and nanoparticle tracking analysis (NTA). Validation involved antibody-based tests (TSG101, CD9, CD81, CD63). Infrared spectra of small EVs were obtained, revealing significant differences in lipid acyl chains, particularly in the C–H stretching of CH3. The study focused on the lipid region (3050–2900 cm^−1^), identifying peaks (3015 cm^−1^, 2960 cm^−1^, 2929 cm^−1^) as distinctive lipid characteristics. Spectroscopic lipid-to-lipid ratios [(I3015/I2929), (I2960/I2929)] emerged as prominent breast cancer markers. Exploration of protein, nucleic acid, and carbohydrate ratios indicated variations in alpha helices, asymmetric C–H stretching vibrations, and C–O stretching at 1033 cm^−1^. Principal component analysis (PCA) successfully differentiated BrCa and HC small EVs, and heatmap analysis and receiver operating characteristic (ROC) curve evaluations underscored the discriminatory power of lipid ratios. Notably, (I2960/I2929) exhibited 100% sensitivity and specificity, highlighting its potential as a robust BrCa sEV marker for breast cancer detection.

## Introduction

Breast cancer, recognized as the predominant malignancy affecting women globally, necessitates a sophisticated approach to early detection and diagnosis. Despite survival rate variations, the swift identification and intervention significantly improve overall prognoses, highlighting the pressing need for advanced diagnostic methodologies^1–3^.

While traditional detection methods like breast exams, mammography, ultrasounds, histological analyses, and magnetic resonance imaging are invaluable, their limitations in addressing the diverse spectrum of breast cancer subtypes and providing comprehensive diagnostic accuracy underscore the urgency for novel diagnostic approaches^4^. Thus, there is a critical need for pioneering early breast cancer diagnostic methods to advance diagnostic research and enhance clinical outcomes.

Invasive tissue sampling for cancer detection poses multifaceted challenges, including patient discomfort, procedural risks, and limitations in accessing diverse or critical tissue regions. These challenges hinder the frequency of cancer assessments, making early, definitive diagnoses more challenging. In this context, liquid biopsy emerges as a promising alternative, focusing on analyzing nonsolid biological material, particularly blood. By offering noninvasive or minimally invasive procedures, liquid biopsy enables frequent testing, contributing to efficient disease diagnosis and the assessment of treatment effectiveness^5^.

While both exosomes and small extracellular vesicles (sEVs) play pivotal roles in intercellular communication and disease pathogenesis, focusing specifically on small EVs offers a nuanced perspective. These tiny vesicles harbor intricate lipid profiles that may hold diagnostic significance in breast cancer and other conditions. Extracellular vesicles (EVs) play a pivotal role in both physiological and pathological conditions. sEVs are tiny, lipid-bound structures which are released by various cell types and carry a diverse cargo, including lipids, proteins, nucleic acids, and glycoproteins. Among their intriguing features is the formation of an “sEVs corona,” which consists of these components and significantly influences sEVs aggregation, biodistribution, clearance, and other biological properties. Importantly, this sEVs corona holds promise as a potential source of valuable “biomarkers” for clinical analysis. Recent research, such as the study conducted by Stępień and colleagues, sheds light on the multifaceted roles of sEVs in vascular pathophysiology. In this review, we delve into specific characteristics of sEVs that remain relatively unexplored but contribute significantly to their biological activity. Let’s explore the fascinating world of EVs beyond their molecular content^6,7^.

Fourier transform infrared spectroscopy (FTIR), particularly in attenuated total reflection (ATR) mode, is a viable method for the label-free chemical profiling of small EVs, as evidenced by the publication of numerous papers on the subject^8–10^. Most of this research employs in vitro procedures to determine sample composition and purity^11–13^, classify various EVs^14^, or identify EVs produced by cells at different developmental stages, phenotypes, or culture conditions^15^. EVs extracted from participants' biofluids in clinical investigations have yet to be the topic of many FTIR studies. These articles include Yap et al.’s description of EVs purified from patients with prostate cancer’s urine and Zlotogorski-Hurvitz et al.’s investigation of salivary EVs from breast cancer patients^16–18^. To our knowledge, no research on breast cancer has yet been published. This research focuses on analyzing FTIR spectra of breast liquid biopsy samples and reporting and comparing findings for breast cancer patients and controls to gain insights into disease progression.

## Result

### Study groups

The subjects in the study were divided into two groups: breast cancer patients (BrCa) and healthy controls (HC). The HC group had 30 females aged between 21 and 53 years, and the BrCa group had 75 females aged 23 to 51 years. The females selected were in the early stages of the disease (T_1-2_/N_0_M_0_) (Table 1).

**Table 1:** F—female; ER—estrogen receptor, PR—progesterone receptor, Her2neu—human epidermal growth factor receptor 2.

Study group	Age	Gender	Clinical stage	Receptor status—positive
Healthy Controls	21–67	F	–	–
Breast cancer	22–71	F	1–2	ER, PR, Her2neu

### Characterization and validation of plasma-derived small EVs (sEVs)

**Figure 1:**
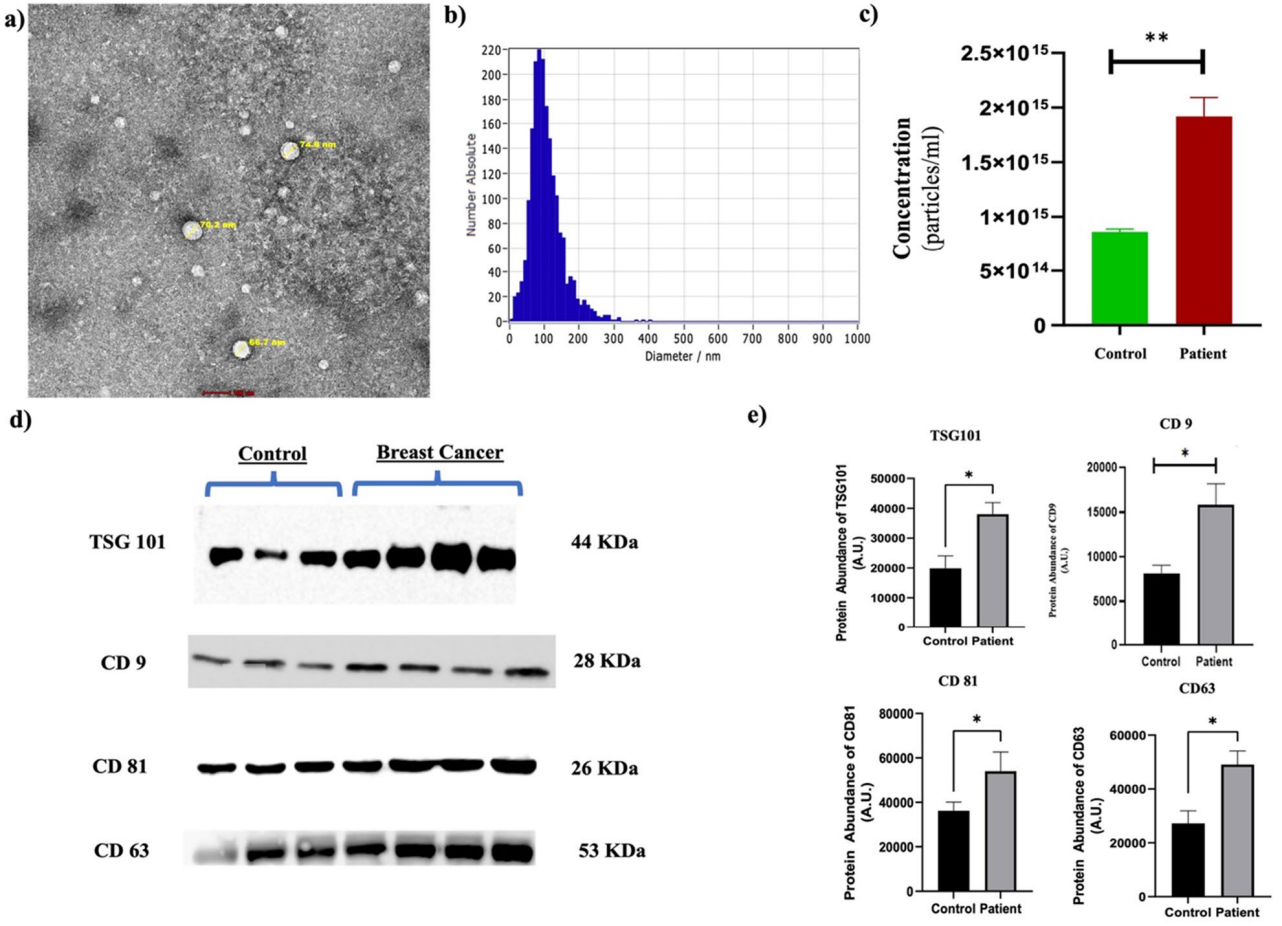
Characterization and validation of isolated plasma sEVs. (**a**) Morphological characterization of isolated plasma sEVs through transmission electron microscopy (scale bar 100 nm) (**b**, **c**) NTA result for size distribution of plasma sEVs; Bar graph of control and patient. (**d**, **e**) Western blot of TSG101, CD9, CD81 and CD63 for sEVs validation; Densitometric analysis of Western blot of TSG101, CD9, CD81 and CD63.

Transmission electron microscopy (TEM) was employed for the characterization of sEV morphology (Fig. 1a). A representative TEM image of isolated plasma sEVs has a scale of 100 nm, and sEVs are round/spherical in shape and feature a lipid bilayer on the outside. The average number was slightly greater when comparing patient sEVs with the control. (Fig. S1). The NTA representative image in Fig. 1b shows the size distribution of all sEVs, and the mean size of plasma-derived sEVs is close to 100 nm. Most of the particles in the sample ranged from 30 to 150 nm. On comparing the NTA of isolated sEVs of HC and BrCa, there was a significant increase in the concentration of BrCa patient to Control (Fig. 1c). Furthermore, the plasma-derived sEVs were validated by Western blotting with the well-known sEVs surface markers CD9, CD81, CD63 and TSG101 (Fig. 1d). The densitometric analysis represents significantly increased expression of sEVs marker proteins in breast cancer patients compared to the control group, which validates the NTA and TEM data. (Fig. 1e).

### Analysis of plasma derived sEVs infrared spectra

**Figure 2:**
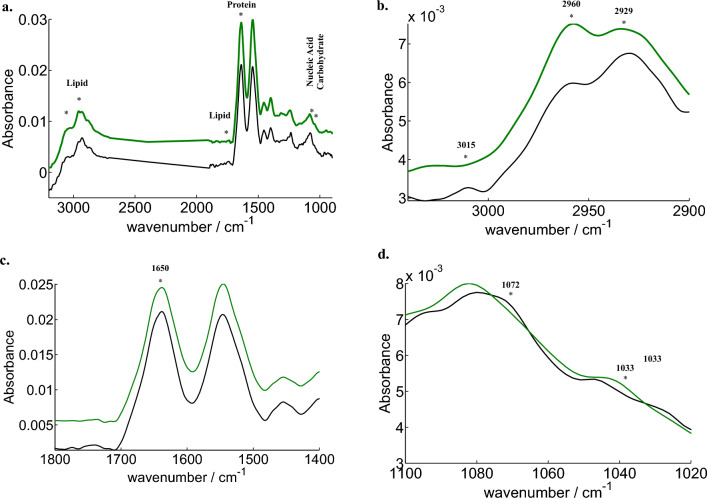
Full length representative spectra from 3100 to 900 with green symbolizing Patient and black as Control, star shows the visible difference; (**a**) spectra with area with oxidative stress (=CH, 3015 cm^–1^), lipids (3000–2800 cm^–1^) (**b**) 1700–1500, amide I (1650 cm^–1^), (**c**) 1090–1030, nucleotides (1072 cm^–1^) carbohydrates (1033 cm^–1^),^24^.

FTIR shows molecular-vibrational transitions, which also offers distinctive details on molecular compositions that could decipher the sEVs composition. Figure 2a displays the sEVs absorbance for both the control and patient (black and green, respectively). The visible variations between two are observed in the lipid and in some areas of protein, nucleic acids, and carbohydrate regions, with a star indicating the spectral region considered, spanning from 900 to 3100 cm^−1^. This initiates the hunt for a spectral marker for the early diagnosis of disease. According to published studies^19,20^, the specific description of spectral characteristics of cellular components for the FTIR spectrum of a cellular sample can frequently be divided into a number of spectral ranges, each of which corresponds to a particular cellular component.

#### Analysis of lipids changes

The importance of lipids in sEVs is linked to many diseases; the acquired spectra unveiled a significant difference in lipid areas absorption (resulting shift) between healthy individuals and patients, emphasizing the potential of IR spectroscopy to discern alterations in lipid profiles. Given the capacity of IR spectroscopy to simultaneously assess various lipid stretching components, we opted for a ‘spectroscopic lipid-to-lipid ratio’ to facilitate a comprehensive and unbiased analysis. While normalization of all peaks in a uniform manner could be an alternative, the absence of a specific universally consistent peak across sEVs samples prompted us to employ ratioing to mitigate potential artifacts arising from concentration variations. The importance of lipids in sEVs is linked to many diseases; here, in healthy and patient breast cancer sEVs is addressed in Fig. 2b and Fig. S2 by comparing three prominent areas corresponding to the lipid region between the two groups: 3050–3000 cm^−1^ (=CH olefinic group), 3000–2800 cm^−1^ (CH_2_ and CH_3_ stretching vibrations among lipid acyl chains), and 1760–1720 cm^−1^ (of carbonyl ester)^21^. A=CH vibration (olefinic, degree of saturation in fatty acids) is attributed to the band at 3015, Asymmetric stretching vibrations of =C–H bond in the methyl (CH_3_) groups of lipids are observed at 2960 band, 2929 band corresponds to asymmetric stretching vibrations of the =C–H bond in the methylene (CH_2_) groups of lipids^22^.

**Figure 3:**
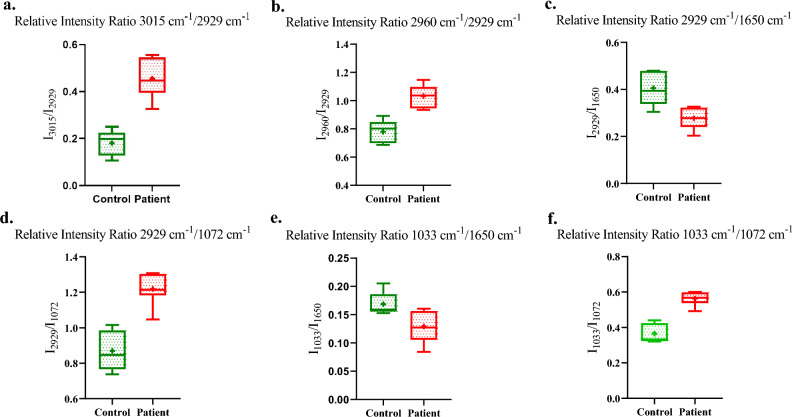
(**a**) Relative intensity ratio I_3015_/I_2929_. (**b**) Relative intensity ratio I_2960_/I_2929_. (**c**) Relative intensity ratio I_2929_/I_1650_. (**d**) Relative intensity ratio* I*_2929_/I_1072_. (**e**) Relative intensity ratio* I*_1033_/*I*_1650_. (**f**) Relative intensity ratio* I*_1033_/*I*_1072_.

Employing a meticulous approach to minimize errors, we calculated the ratios (I_3015_/I_2929_) and (I_2960_/I_2929_), focusing on these prominent markers for the disease^13,23^. The absorbance ratio at I_3015_/I_2929_ in patients was significantly higher (P < 0.05) than that of healthy subjects, demonstrating a mean difference of 0.2741 ± 0.04. A parallel trend was observed at I_2960_/I_2929_, with a mean difference of 0.2530 ± 0.04 (Fig. 3a and b). These findings underscore the robustness of the spectroscopic lipid-to-lipid ratio as a valuable metric for comparative analysis, revealing distinct lipidomic signatures associated with pathological conditions.

#### Other biomolecules (proteins, nucleic acids and carbohydrates) changes

Proteins, as pivotal biomolecules, exhibit nuanced differences in their absorption bands, as depicted in Fig. 2c. A comprehensive assessment of the total protein content in patient sEVs revealed a distinctive protein profile characterized by a diminished presence of alpha helices and an increased prevalence of random twists compared to healthy sEVs (Figs. S3, S4, and Table S1)^24^. An alternative approach involves the examination of asymmetric C–H stretching vibrations in fatty acids relative to amide I, providing insights into the composition of membrane proteins in biological membranes and confirming that the changes are from lipid structures of sEVs. The ratios of the acyl band to the amide I band (I2929/I1650) demonstrated a significantly elevated (P < 0.05) relative intensity ratio in Healthy Controls (HC) compared to Breast Cancer (BrCa) patients, with a mean difference of 0.1282 ± 0.03 (Fig. 3c).

Another salient band observed at 1072 cm^−1^ informs about the symmetric phosphate stretching of nucleic acids, enriching the spectral information in the fingerprint region (Fig. 2d). Notably, BrCa spectra exhibited distinct differences from those of healthy subjects in the range of 1100–1000 cm^−1^. Calculating the ratio of the acyl band (lipid) to symmetric phosphate (nucleic acid), BrCa manifested a significantly higher (P < 0.05) relative intensity ratio for the band ratio (I_2929_/I_1072_), with a mean difference of 0.3486 ± 0.05 (Fig. 3d). Further spectral analysis uncovered variations in the C–O stretching band at 1033 cm^−1^ (carbohydrate) (Fig. 2d). Relative intensity comparisons with amide I at 1650 cm^−1^ revealed that HC exhibited a significantly higher (P < 0.05) relative intensity ratio (I_1033_/I_1650_) than patients, with a mean difference of 0.0395 ± 0.01 (Fig. 3e). Alternatively, exploring the carbohydrate to nucleic acid ratio, BrCa displayed a significantly greater (P < 0.05) relative intensity ratio (I_1033_/I_1072_) than HC, with a mean difference of 0.1967 ± 0.02 (Fig. 3f). These intricate spectral nuances unravel distinct molecular signatures, shedding light on the complicated biochemical alterations associated with Breast Cancer.

#### PCA plot, heatmap and ROC curve analysis of the changes

**Figure 4:**
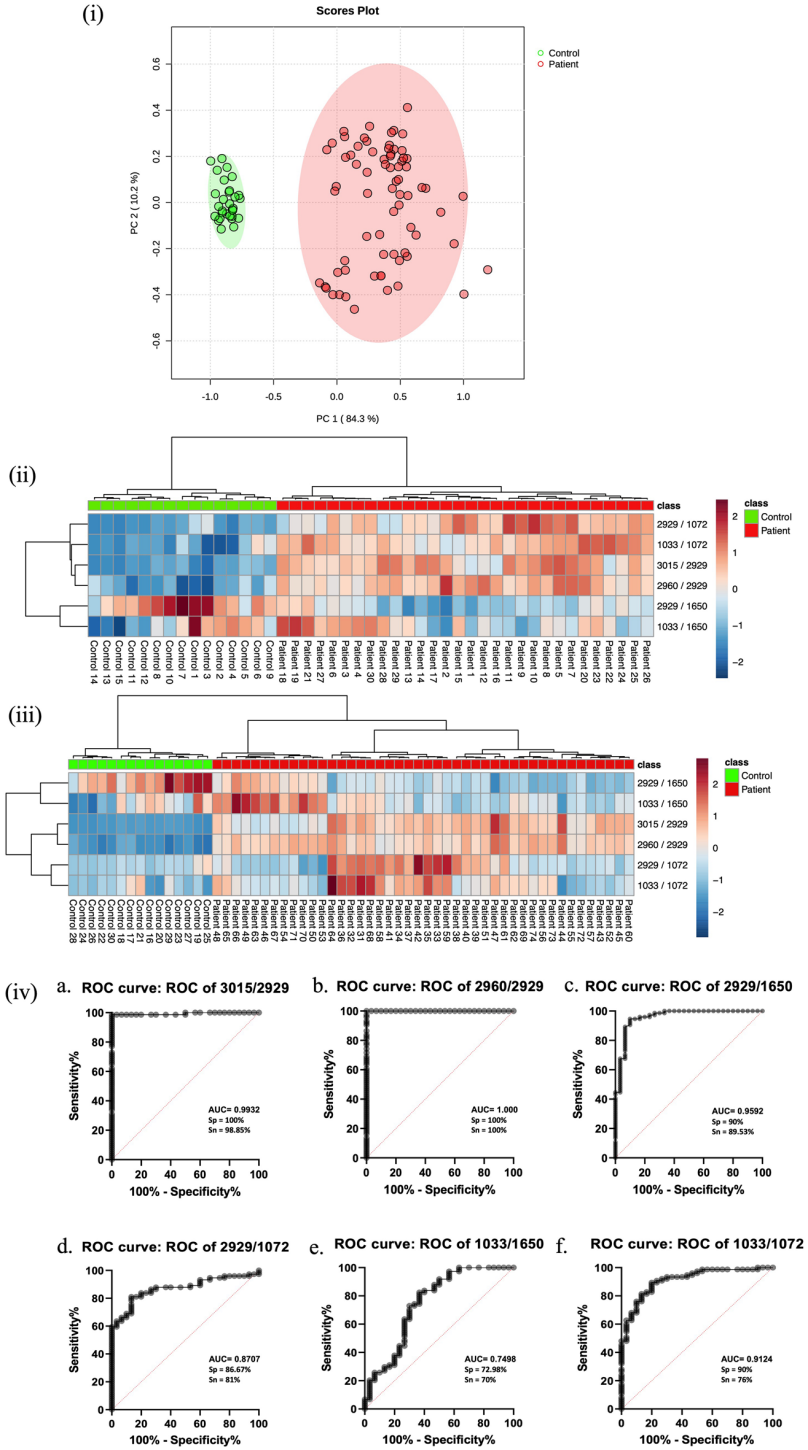
(**i**) Analysis of controls (n = 30) and patients (n = 74) by PCA plot between HC and BrCa sEV spectral ratios. (**ii**) Heatmap of patient (n = 30) and control (n = 15) data per cluster. Bars on the right show the color scale representing the proportion of patients and controls with each characteristic regarding respective ratios. (**iii**) Heatmap of test samples of patient (n = 44) and control (n = 15) data per cluster. For continuous variables, it represents a scaled value from highest cluster mean (2.0) to lowest cluster mean (− 2). (**iv**) ROC curve with AUC, specificity, and sensitivity of all the respective ratios.

In our comprehensive analysis, we harnessed principal component analysis (PCA) to meticulously scrutinize the discriminative potential residing within specific absorbance band ratios, covering the spectral range from 900 to 3100 cm^−1^. This intricate examination allowed us to unravel nuanced biochemical alterations, as depicted in Fig. 4i. The input for PCA was carefully curated, incorporating ratios derived from pivotal bands such as (I_3015_/I_2929_), (I_2960_/_I2929)_, (I_2929_/I_1650_), (I_2929_/I_1072_), (I_1033_/I_1650_), and (I_1033_/I_1072_) (refer to Table 2 for details).

**Table 2:** The following table represents different band ratios and the biomolecules associated with them and a quick look at which ratio is higher in patients or controls.

Band ratio	Biomolecules which taken into consideration	Higher
3015 cm^−1^/2929 cm^−1^	Olefinic Membrane Lipids/Membrane Lipids acyl chain (CH2)	Patients
2960 cm^−1^/2929 cm^−1^	Membrane Lipids (CH3 a.s.)/Membrane Lipids (CH2 a.s.)	Patients
2929 cm^−1^/1650 cm^−1^	Membrane Lipids (CH2 a.s.)/Proteins (Amide I)	Control
2929 cm^−1^/1072 cm^−1^	Membrane Lipids (CH2 a.s.)/Carbohydrates (C–O str, C–O bend)	Patients
1033 cm^−1^/1650 cm^−1^	Carbohydrates (C–O str, C–O bend)/Proteins (Amide I)	Control
1033 cm^−1^/1072 cm^−1^	Carbohydrate band (C–O str, C–O bend)/Nucleic Acid band	Patients

The discriminant PCA plot, presented in Fig. 4i, is a visual testament to the successful differentiation between sEVs pellets from Breast Cancer (BrCa) patients and Healthy Controls (HCs). Each data point on the score plot, representing a ratioed absorption, underscores the critical role played by these ratios in distinguishing between HC and BrCa plasma sEVs. The green circles elegantly represent HCs, while the red circles depict patients. The segregation along the first component axis (capturing 84.3% variation) and the second component axis (capturing 10.2% variation) elucidates the robust differentiation achieved for HC and BrCa plasma sEVs.

To fortify the diagnostic potential of these ratios, we employed a heatmap and ROC curve analysis (Fig. 4ii and iii). The heatmap, illustrating the intensity corresponding to the ratios of spectral bands for 15 samples and 30 patients, serves as a visual representation reinforcing our ability to discern between healthy and patient groups. A subsequent analysis involving 15 controls and 44 patients reaffirms the consistency of the lipid area in facilitating differentiation. The ROC curve analysis, performed for spectra ratios (Fig. 4iv), provides a quantitative measure of the diagnostic prowess inherent in these ratios. Notably, the I_2960_/I_2929_ band ratio emerged as the most potent discriminator, achieving 100% sensitivity and specificity with an AUC of 1.000. Furthermore, the ratio I_3015_/I_2929_ exhibited remarkable specificity and sensitivity of 100% and 98.85%, respectively, with an AUC of 0.9932. This reaffirms the capacity of these ratios to effectively differentiate BrCa patients from Healthy Controls based on lipid changes.

The prominence of distinct lipid changes in these findings underscores the robust classifier role of the I2960/I2929 band ratio. It provides additional validation for the diagnostic potential inherent in lipidomic alterations within BrCa plasma sEVs.

## Discussion

The study presented here offers a groundbreaking exploration into the complex world of plasma small extracellular vesicles (sEVs) using Fourier-transform infrared (FTIR) spectroscopy, shedding light on its potential for early breast cancer detection. By employing advanced techniques like Principal Component Analysis (PCA) and heatmap visualization, the research team was able to precisely differentiate between sEVs from Breast Cancer (BrCa) patients and those from Healthy Controls (HCs). These distinctions, rooted in the subtle alterations of lipids, proteins, nucleic acids, and carbohydrates, underscore the promise of FTIR spectroscopy in noninvasive cancer diagnostics^20–25^. The noninvasive nature of this approach holds promise for frequent plasma assessments as a measure of clinical outcomes or during follow-up periods, especially in high-risk patient cohorts.

In our methodology, we initially utilized a chemical isolation method with polyethylene glycol (PEG) coupled with Western Blot for validation, Nanoparticle Tracking Analysis (NTA) for size distribution and Transmission Electron Microscopy for morphology. While acknowledging the merits of alternative techniques like differentiation centrifugation and tunable resistance pulse sensing (TRPS) proposed by Durak-Kozica et al., we opted for simplicity and accessibility in our approach. However, we recognize the importance of considering factors such as purity, speed, and precision when choosing isolation and characterization methods, and integrating these discussions enhances the transparency of our methodology. The use of mass spectrometry in protein profiling has become pivotal in biomarker discovery, especially in cancer research^26^.

In cancer biomarker research, attention has focused on CH3/CH2 ratios and lipid oxidation patterns identified through FTIR spectroscopy in tissues and cells^22^. These alterations serve as potential indicators of cancer progression, deepening our understanding of cancer biology and paving the way for innovative diagnostic approaches^27–29^. Extracellular vesicles (EVs), particularly small EVs (sEVs), reflect cellular changes and exhibit unique spectral fingerprints associated with different cancer types, indicative of membrane lipid alterations induced by oxidative stress^30,31^. These observations underscore the potential of FTIR spectroscopy in elucidating cancer-related molecular signatures and the metabolic demands of malignant cells, including Breast Cancer (BrCa) sEVs^20–22^.

Our meticulous spectral analysis, involving background subtraction and ratio calculations, revealed distinct biomolecular alterations in BrCa sEVs compared to HCs. The ratios of specific spectral bands (I_2960_/I_2929_) emerged as potential hallmarks for distinguishing BrCa from HC plasma sEV samples, supported by robust statistical analyses such as PCA and Receiver Operating Characteristic (ROC) curve analysis. The ROC curve analysis of band all the ratios was done where I_2960_/I_2929_ has the best sensitivity and sensitivity reading to 100% with AUC 1.000 whereas similar where lipid other band ratio, I_3015_/I_2929_ has second best specificity and sensitivity of 100% and 98.85% with AUC 0.9932 able to differentiate BrCa patient and Healthy control based on lipid changes. The 2960 cm^−1^/2929 cm^−1^ ratio, validated by PCA and ROC analyses, emerges as a promising tool for BrCa detection.

## Conclusion

We demonstrated for the first time that the lipid signature of plasma-derived sEVs could be potent in following breast cancer progression. The current findings are significant because they demonstrate that cancer sEVs can be accurately distinguished from their healthy counterparts by detecting subtle changes in lipid oxidation. Within the existing framework that integrates sEVs, liquid biopsy diagnostics, and FTIR, this could play a major role in developing next-generation approaches for the early diagnosis of breast cancer.

## Experimental methods

### Subject recruitment, sample collection and processing

Female patients who received a breast cancer (BrCa) diagnosis (n = 74) between 2022 and 2023 at the Institute Rotary Cancer Hospital, IRCH Department, AIIMS, India, were included in the study as per the inclusion criteria. The All Institute of Medical Sciences in New Delhi, India, institutional ethics committee granted ethical clearance. This study's ethical clearance number is IECPG-783/22.12.2022. After obtaining written informed consent, all the subjects were chosen for the study. A comprehensive written participant information sheet and participant informed consent form were provided to the subjects who decided to take part in this investigation, and their signatures were obtained. All procedures used in this investigation were strictly adhered to the relevant standards and regulations by the ethical committee.

Plasma was drawn from BrCa patients before the start of any anticancer therapies. Each patient chosen for HC underwent a careful assessment to rule out the presence of any clinical abnormalities. Immediately after collection, the plasma samples were centrifuged at 2500 × g for 20 min at 4 °C to remove macromolecules. After centrifuging the clear plasma samples at 10,000 × g for 20 min at 4 °C, the supernatant was collected. The experiment included 104 samples, including 74 plasma samples from BrCa patients and 30 samples from the HC group, and all the experiments were run in triple replicates. All sample supernatants were stored until usage at 80 °C.

### Small extracellular vesicles isolation

Using chemical precipitation, sEVs were isolated from BrCa patient and healthy control plasma samples. Centrifuged plasma samples were added to 14% PEG (polyethylene glycol), vortexed, and allowed to sit at 4 °C for 6–8 h to eliminate any substantial impurities. Using 13,000 g, the mixture was centrifuged for an additional hour at 4 °C. Following this process, a pellet was observed that was washed twice and then resuspended in 1× phosphate-buffered saline (PBS). Using centrifugal filters with a 100 kDa cutoff, the resultant suspension was further filtered. Using a chemically based precipitation technique, high-yield sEVs were generated, and a filtration procedure ensured purity and homogeneity.

### Transmission electron microscopy (TEM)

Transmission electron microscopy was used to examine the ultrastructural characteristics of isolated sEVs. A phosphate buffer solution with a pH of 7.4 was used to dilute the sEV pellet. A 300 mesh carbon-coated copper grid (Ted Pella-01843) was used for the adsorptive process, which was completed for half an hour at room temperature using separated sEVs. Using a 2% aqueous uranyl acetate solution, the grids were coloured for 30 s after being blotted dry. The grids were then blot dried before being examined using an FEI, USA, transmission electron microscope.

### Nanoparticle tracking analysis

Briefly, approximately 0.5 ml of diluted sEV sample was loaded into the sample chamber of the Zeta View Twin system. Three cycles were performed by scanning 11 cell positions each and capturing 60 frames per position (video setting: high) under the following settings: Focus: autofocus; Camera sensitivity for all samples: 80.0; Shutter: 150; Scattering Intensity: 5.0; embedded laser at 488 nm; Cell temperature: 25 °C. The videos were captured using a CMOS camera and analyzed by the in-build ZetaView Software 8.05.12 with specific analysis parameters: maximum particle size: 1000, minimum particle size 10, minimum particle brightness: 30. NTA facilitates the analysis of the size and concentration of particles in solution. All plasma-isolated sEV samples were quantified using NTA at 1:1000 dilutions in 1×-PBS buffer.

### Western blotting (WB)

The ready samples (10 μl) were loaded with loading dye and then subjected to 8–12% SDS‒PAGE. Using the Bio-Rad western blotting apparatus, wet-mode western blotting was carried out on the produced gel. 3% BSA in Tris-base saline added to 0.1% Tween 20 (TBST) was prepared to block the proteins after they had been transferred from the gel to the 0.22 PVDF membrane. Primary antibodies against CD63 (10628D, Invitrogen), CD81, CD9, and TSG101 were incubated overnight at a dilution of 1:5000 in 1.5% BSA in TBST. Eventually, the blot was located utilizing the enhanced chemiluminescence (ECL) detection method from GE Healthcare Buckinghamshire.

### Data acquisition and processing through FTIR

A concentrated sample (4 μl) was kept in an ATR reflection diamond element. A DTGS detector and an Agilent Cary 600 FTIR instrument with a resolution of 4 cm^−1^ were used to collect FTIR spectra. A liquid sample was used for the experiments carried out at room temperature using Agilent ResolutionPro software. A single beam spectrum of the buffer (PBS) in which each sample was resuspended was used to obtain the background spectrum for 128 scans for each sample. There was continuous purging the instruments using dry air^24^.

We evaluated the spectral properties of freshly isolated samples in liquid form. Before measuring a new sample, an initial background measurement was performed (PBS was applied to the diamond ATR crystal and taken spectra as the reference). Immediately following the sample’s measurements were made at room temperature. The spectral wavenumbers was from 900 to 3100 cm^−1^, with a nominal resolution of 4 cm^−1^. For all measurements, 128 scans were acquired to improve the signal-to-noise ratio. GraphPad Prism, Agilent Resolution Pro, and Erik Goormaghtigh’s MATLAB (Mathworks, Natick, MA, USA) program, Kinetics, were used to process the data.

The second derivative spectra were computed after applying a 4 cm^−1^ smoothing using the Matlab program Kinetics, authored by Erik Goormaghtigh^32^. These spectra were carefully examined for the absence of water vapor features, particularly above 1750 cm^−1^. The spectra showcased in the figures underwent normalization to ensure uniform band amplitudes. For the secondary structure estimation, curve fitting of the amide I region was conducted employing Kinetics. Preceding the curve fitting process, a straight baseline was subtracted, passing through the ordinate at 1700 and 1600 cm^−1^. The baseline underwent further adjustments through a least-squares curve-fitting program, allowing for fine-tuning a horizontal baseline as an additional parameter to achieve the optimal fit. The determination of initial peak positions for curve fitting was facilitated by a second derivative spectrum, and the actual curve fitting process utilized Voigt functions^33^.

### Spectral preprocessing method

Preprocessing procedures were implemented to enhance the fidelity of all FTIR spectra. The Savitsky-Golay smoothing method, employing a window width of five points, was judiciously applied to each spectrum to mitigate the impact of random noise^34^. The baseline correction of spectral data was conducted employing a rigorous six-point correction methodology. Specifically, distinct wavenumbers at 3200 cm^−1^, 2700 cm^−1^, 1950 cm^−1^, 1485 cm^−1^, 1000 cm^−1^, and 801 cm^−1^ were strategically chosen as reference points for the correction procedure. This meticulous approach was designed to rectify and standardize baseline fluctuations inherent in the spectral data, aiming to impart precision and consistency in the representation of the underlying chemical information. Subsequently, a thorough visual analysis ensued to discern absorbance bands manifesting significant distinctions between the experimental groups. This multi-step process contributes to the enhancement of data accuracy and reliability in the context of spectral analysis. Calculations of height ratios for these bands ensued, and a t-test was employed to ascertain the significance of any observed differences among the groups, with a predetermined significance threshold of P < 0.05. This robust preprocessing protocol ensures the reliability and accuracy of the subsequent spectral analyses. More details related to PBS challenges are added in the Supplementary.

### Statistical analyses

All statistical analyses were performed utilizing GraphPad Prism 8.0 software. Unpaired Student’s t test results were used to calculate statistical significance. P < 0.05 was used to determine significance throughout. With the help of a multivariate statistical method called PCA (Principal Component Assay), it is possible to examine multiple parameters simultaneously. When several different parameters are to be assessed, they are employed in collective characterization. Because it permits a wider perspective than just taking the location and intensity of spectral properties into account, PCA is frequently utilized among pattern recognition as well as in spectroscopy. Few variables from a large collection of data are selected for PCA, which then shows it in a dimensional space of uncorrelated principal components, which are linear combinations of the original variables^35^.

The whole area under the ROC curve (receiver operating characteristic curve), or AUC (area under the curve), is a measure of separability. The ROC is a probability curve used to analyze test accuracy. It indicates the degree to which the test can discriminate between classes. The test's ability to discriminate between people who have a medical condition and those who do not is facilitated by a greater AUC. TPR (sensitivity) is placed on the y-axis and FPR (specificity) is put on the x-axis to create the ROC curve. An AUC that is close to 1, indicating a good measure of separability, is indicative of an excellent test. An AUC close to 0 indicates a bad test, which has the lowest separability measure. Here, ROC analyses were utilized to determine the accuracy of different approach.

### Supplementary Information

Supplementary Information.

## Data Availability

The data supporting this study’s findings are available from the corresponding authors, [SK, AM], upon reasonable request.
