# Supplementary material for: Spectroscopic insight into breast cancer: profiling small extracellular vesicles lipids via infrared spectroscopy for diagnostic precision
Source: Sci Rep. 2024 Apr 23;14:9347. doi: 10.1038/s41598-024-59863-1 (PMC11039614; doi:10.1038/s41598-024-59863-1)
Supplement: Supplementary file 1 — Supplementary Information. [file 41598_2024_59863_MOESM1_ESM.doc]

**Supplementary.**

**Supplementary Figure 1.**


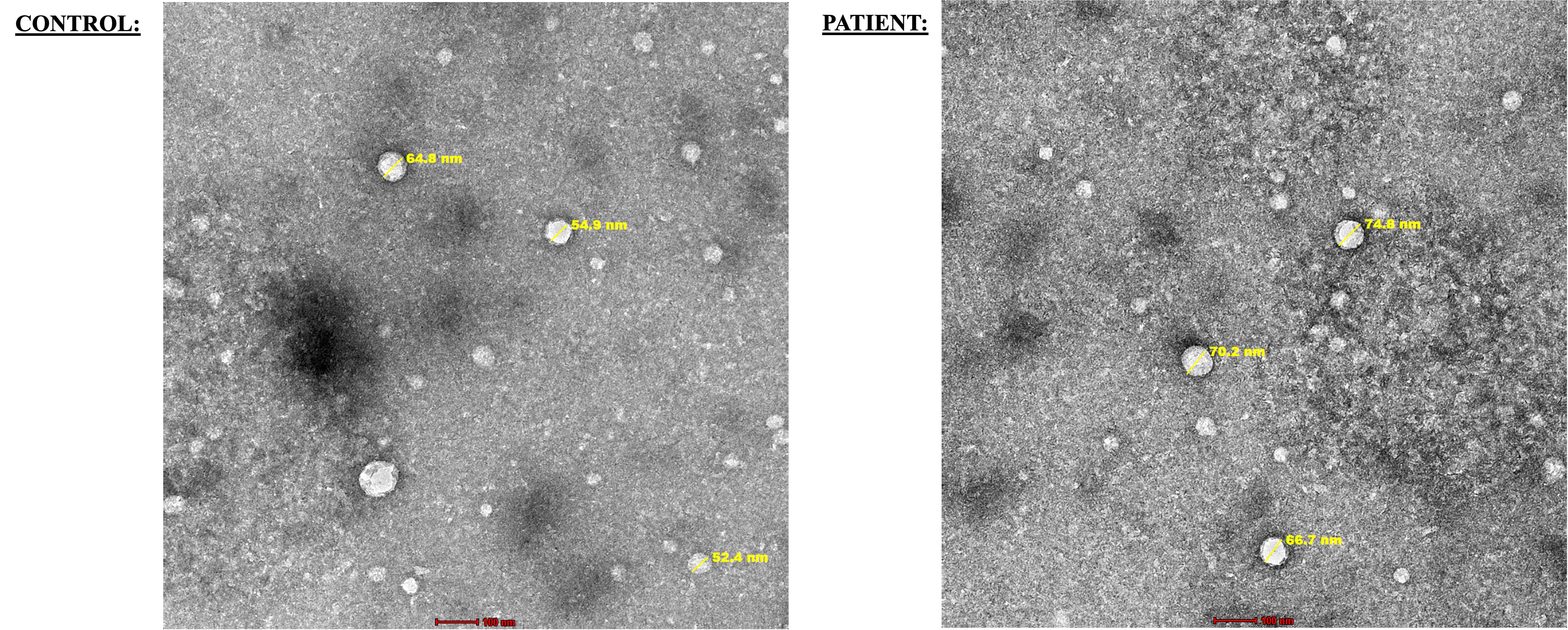


**Figure S1:** TEM images of Control and Patient, the concentration seems to be increased of the Patient sEVs.

**Supplementary Figure 2.**

**Figure S2:** **AVERAGE CARBONYL ESTERS (1760–1720 CM^–1^) ABSORBANCE SPECTRA OF HEALTHY VS PATIENT**. Data are means of individual of Patient and Control.

**Supplementary Figure 3.**

a.

b.

**FIGURE 3. (a**) Curve fitting of the amide i region (1700–1600 cm–1) of the control (b). Curve fitting of the amide I region (1700–1600 cm–1) of the patient.

**Supplementary Table S1.**

Table S1. **TOtal pROTEIN SECONDARY STRUCTURE DATA OF CONTROL AND PATIENT sEVs.**

|  | Alpha (%) | beta (%) | Unstructured/Random coils (%) |
| --- | --- | --- | --- |
| Control | **35.22** | 29.05 | 35.68 |
| Patient | 29.06 | 28.8 | **42.14** |

**Supplementary Figure 4.**

**FIGURE 4.** Differentiated region of amide I in control and breast cancer patient sEVs.

**Western Blots:**

**TSG 101:**


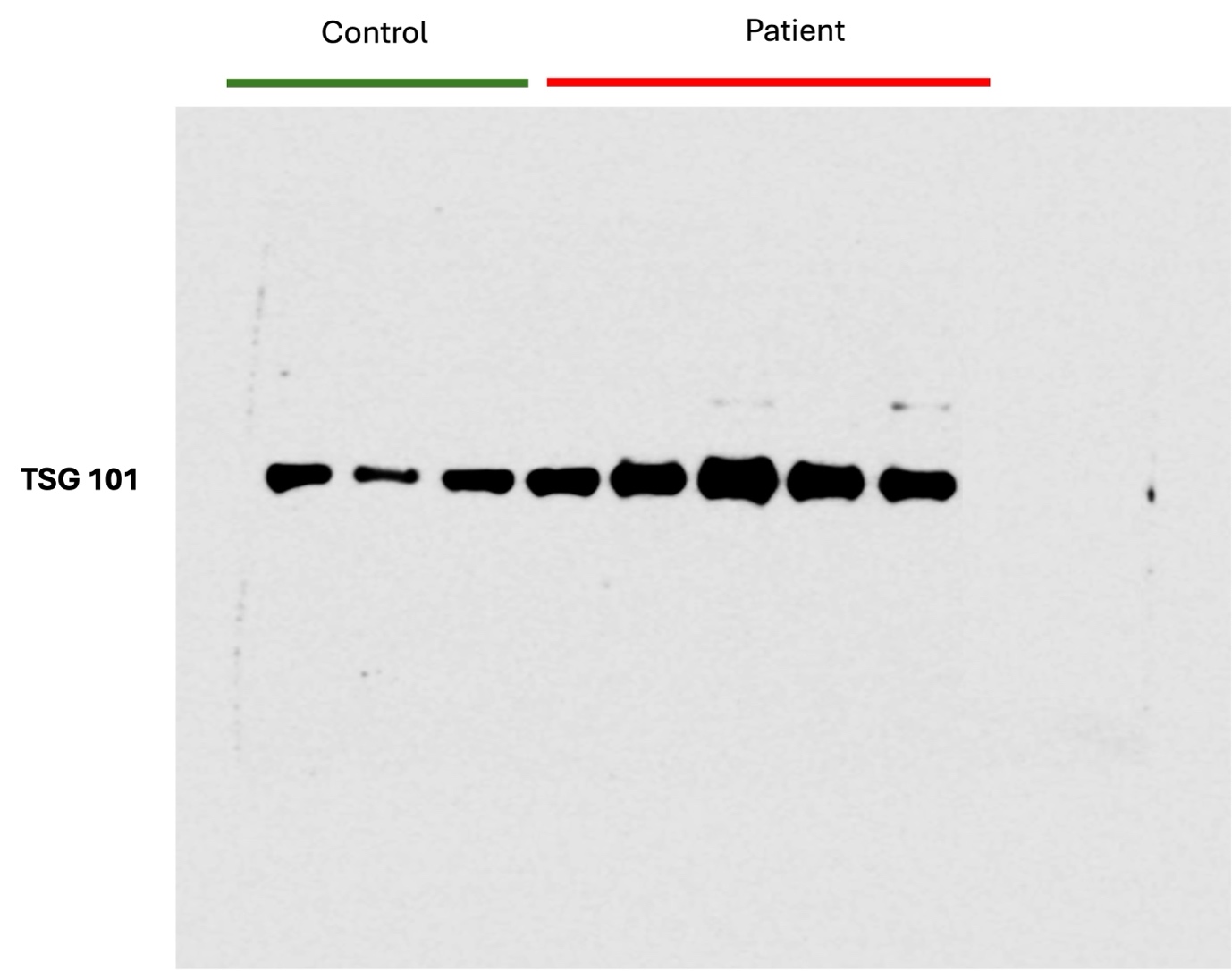


**Figure WB-S1:** **Western blot of anti-TSG 101** **in Healthy Controls (Lane 1,2 and 3), Patient (Lane 4-8).**

**CD9:**


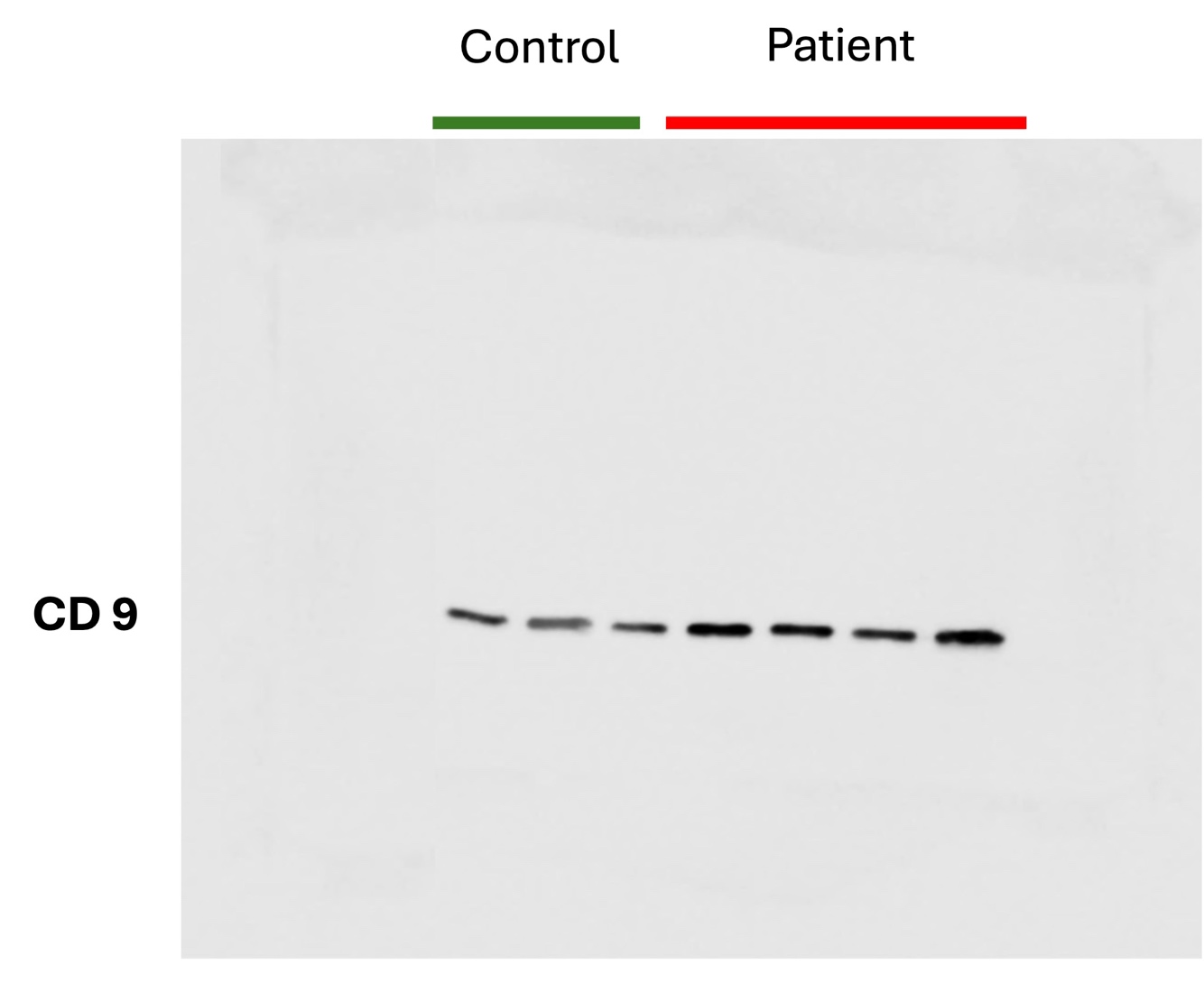


**Figure WB-S2: Western blot of anti-CD 9 in Healthy Controls (Lane 2, 3 and 4), Patient (Lane 5-9).**

**CD81:**


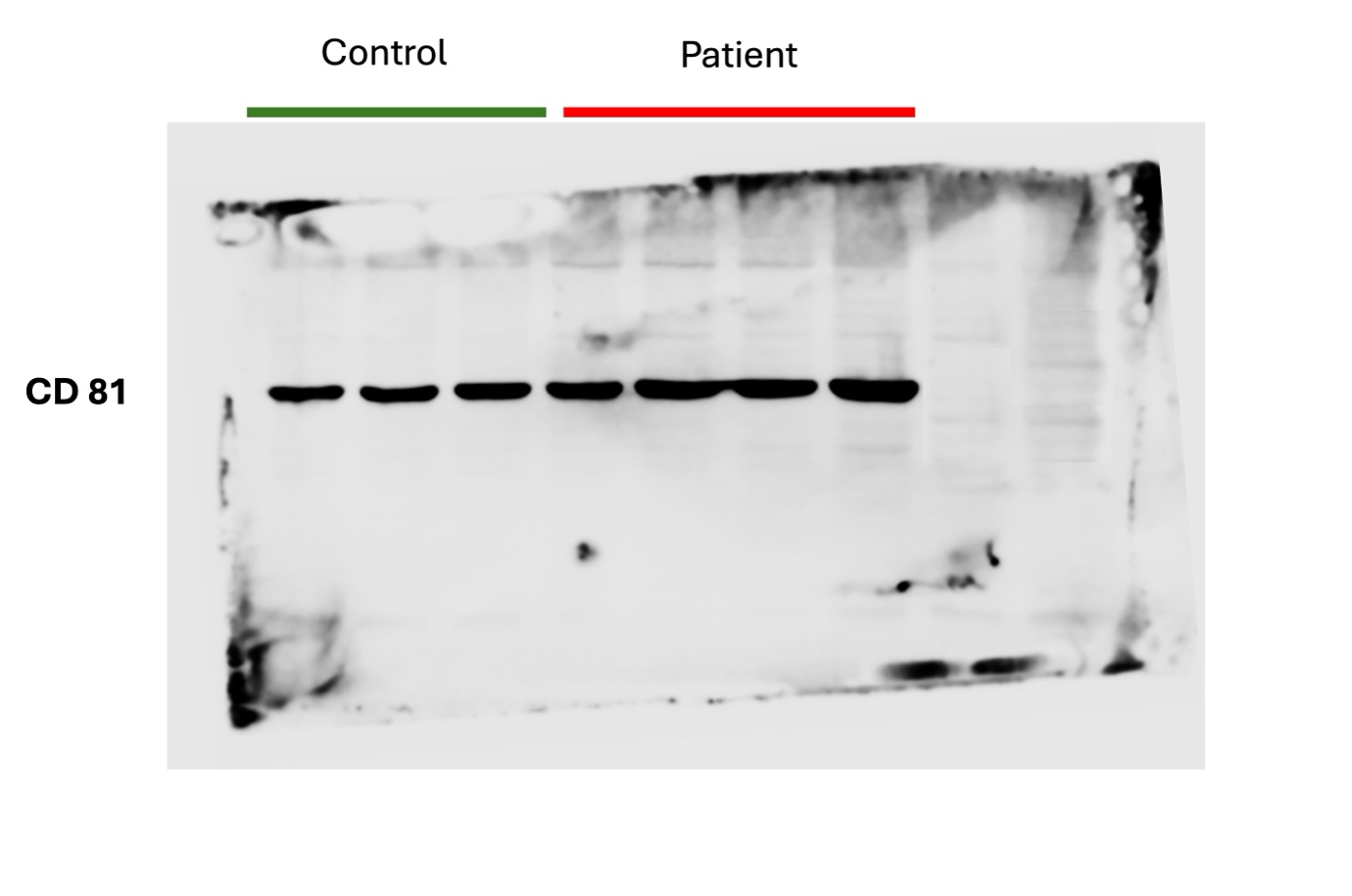


**Figure WB-S3: Western blot of anti-CD 81 in Healthy Controls (Lane 1,2 and 3), Patient (Lane 4-8).**

**CD63:**


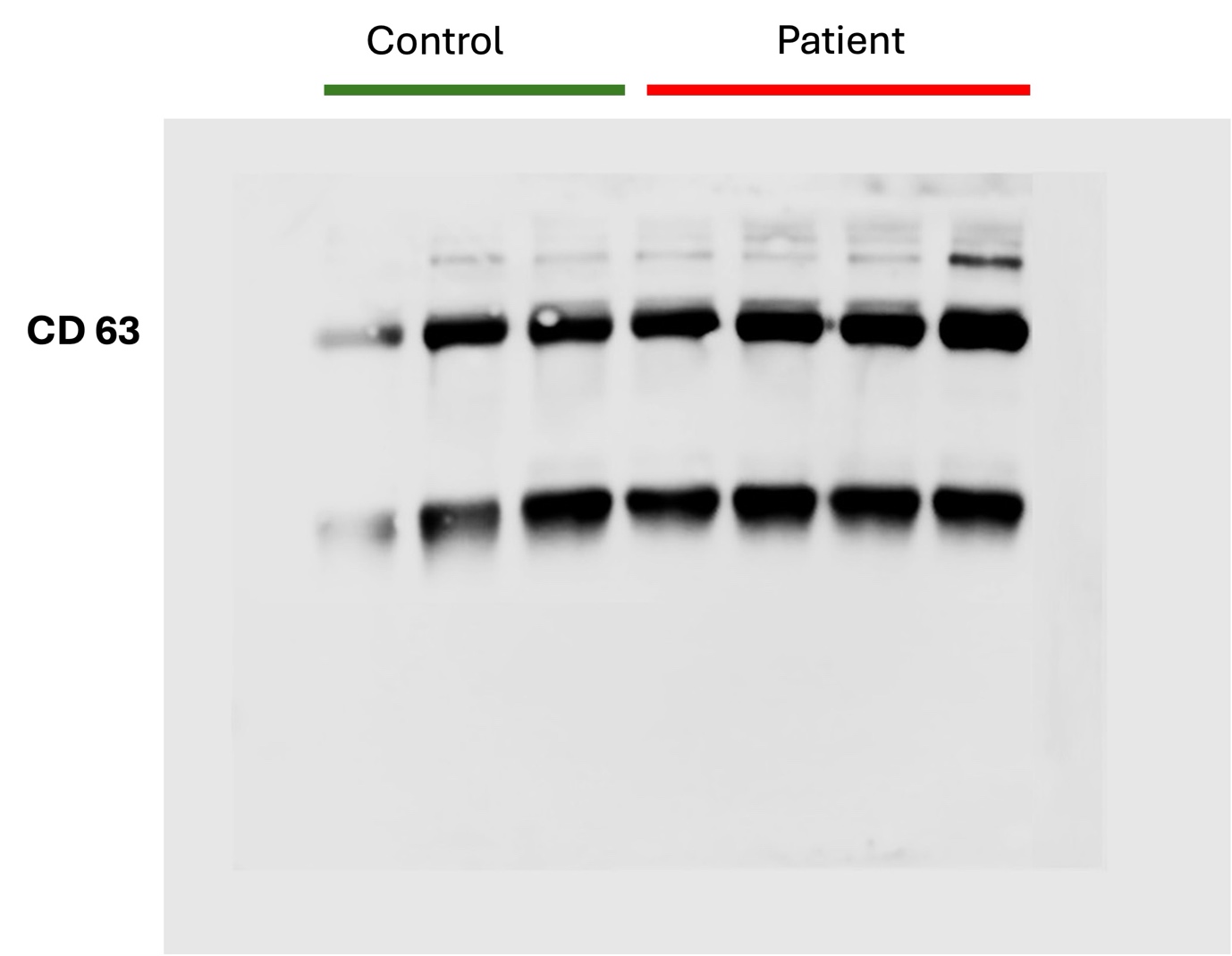


**Figure WB-S4: Western blot of anti-CD 81 in Healthy Controls (Lane 2,3 and 4), Patient (Lane 5-9).**
